# Analysis of real crashes against metal roadside barriers

**DOI:** 10.1371/journal.pone.0211674

**Published:** 2019-02-04

**Authors:** Miguel A. Fernández, Luis Ángel García-Escudero, Aquilino Molinero

**Affiliations:** 1 IMUVA–Departamento de Estadística e Investigación Operativa, Universidad de Valladolid, Valladolid, Spain; 2 Departamento de Ingeniería Energética y Fluidomecánica, Escuela de Ingenierías Industriales, Universidad de Valladolid, Valladolid, Spain; Virginia Tech, UNITED STATES

## Abstract

**Objective:**

Metal Road Safety Barriers (MRSB) are one of the devices implemented in roadsides to mitigate the consequences of run-off crashes. In Europe, they have to meet the requirements of the European Standard EN-1317-2. This article analyzes a set of run-off crashes against MRSB, for which an in-depth investigation has been performed, comparing them with the standard tests. It has been observed that in many of these real crashes, the barriers have not worked properly in spite of having passed these standard tests. This paper demonstrates which variables may be responsible for this, with the objective of helping to improve the current test standard through the analysis of new test variables.

**Methods:**

Multidimensional Scaling, a dimension reduction multivariate statistical technique, has been used to better understand how real crashes compare to standard tests, using several impact variables at the same time. Then, a statistical analysis has been developed to show the influence of the *“Relative orientation impact angle”* on the performance of the MRSB.

**Results:**

Most of the real crashes analyzed are close to “TB11” and “TB32” standard tests. In many of these real crashes, the *“Relative orientation impact angle”* is very different from the *“Impact angle”*, and in these situations, the vehicle is not safely redirected to the road concerning the so-called *“Exit-Box”*.

**Conclusions:**

MRSB are not working properly in some situations that are not far from the standard tests. To handle this, it could be interesting to include the *“Relative orientation impact angle”* as a control variable in new versions of the EN-1317-2 tests to guarantee the behavior of the MRSB. These results can help to adapt some test variables from the EN-1317-2 to what is happening in crashes.

## Introduction

Although around 1.25 million people die yearly on the roads, traffic crashes are predictable, preventable and, in particular, can be mitigated [1]. In most countries around the world, about 30% of all road fatalities are single vehicle crashes [2], where one vehicle leaves the road and strikes a rigid object, rolls over or goes down a steep slope. Statistics illustrate the fact that when it comes to crash severity, run-off-road crashes are an important subset of crashes worldwide and crashes resulting from lane departure constitute a high proportion of severe or fatal crashes. For instance, the average fatality rate for this type of crashes accounted for approximately 1/3 of road deaths in the European Union [3, 4] or 53% of all fatal crashes in the United States [5]. With the aim of mitigating the possible consequences of these single vehicle crashes, Road Restraint Systems (RRS) are installed in roadsides as they are one of the most efficient road infrastructure solutions. For example, the installation of a median barrier on a single carriage way in Israel resulted in a 23% estimated reduction of impacts and 50% estimated reduction in injuries [6], or the existence of RRS may reduce fatalities by a factor of four in France [6], of five in Belgium [6], or of three in some European studies [6] when compared to collisions against other road obstacles.

An RRS is generally a collapsible or sliding structure that, through its deformation or displacement, absorbs part or all of the energy of a vehicle that hits it, and afterwards redirects and/or stops it safely. There are several types of RRS: Road Safety Barriers (RSB), (the most common systems in roads, these are placed along the roadside or on the central reservation with the role of preventing errant vehicles from crashing into roadside obstacles, and to retain them safely), crash cushions, terminals for RSB, motorcycle protection systems and transitions between two RSB [6]. Each of these RRS has to comply with several safety requirements defined in different standards (EN-1317-2 [7] in the European Union (EU) or MASH [8] in the United States of America (USA)) which assess the behavior of the RRS during the impact of a vehicle. This is why crash tests are performed in the standards. The crash conditions established by the standard tests are settled to guarantee the conventional levels of (passive) safety at roadsides, considering the “state-of-the-art” of crash-testing technology, the repeatability of the tests and, of course, the need to assess reliable safety features.

With the aim of improving the “state-of-the-art” in run-off crashes against the most common RRS, metal road safety barriers (MRSB), this article shows information gathered during 12 in-depth crash investigations. In all of them, crash variables (speed impact, impact angle, total weight, vehicle type,…) have been analyzed using reconstruction techniques and compared with test impact requirements defined in one of these specific standards (EN-1317-2), with the objective of helping to improve this current test standard—(or even other similar standards) and helping to prevent injuries in crashes.

## Materials and methods

### Data

Information analyzed in this article comes from two sources:

#### 1.—EN-1317-2 test impact requirements

To implement RRS on roads within the European Union (EU), these systems must meet the requirements of the European Standard EN-1317. In the case of MRSB, which are the most common RRS, EN-1317-2 defines the impact tests to be carried out for assessing them once a vehicle impacts against them. These definitions include the vehicle type to be used—passenger cars, heavy goods vehicles or buses—and how to evaluate whether the impact against these barriers can be considered as “safe” or not based on bio-mechanical parameters measured with sensors and on the deformation of the barrier after the impact. The first part of Table 1 shows the tests detailed in EN-1317-2 to be carried out only with passenger cars (which is the most common vehicle on roads and the one considered in this study). The combination of these tests will be used to give the so-called *“containment level”* to an MRSB. For example, the most common containment level on roads is the “N2 level”, which means that the MRSB complies with tests TB32 and TB11, so this MRSB should be able to contain a passenger car weighing 1,500 kg at 110 Km/h and a passenger car weighing 900 kg at 100 Km/h. This level is what is needed on most non-urban roads.

**Table 1:** (First part) Impact tests for MRSB taking into account passenger car impacts based on standard EN-1317-2 [7] and (second part) characteristics of real crashes analyzed in this study.

Test or Crash number	Impact speed (km/h) (V1)	Impact angle (degrees) (V2)	Total vehicle mass (kg) (V3)	Lateral Kinetic Energy (kJ) (V4)
Test TB11	100	20	900	40.6
Test TB21	80	8	1,300	6.2
Test TB22	80	15	1,300	21.5
Test TB31	80	20	1,500	43.3
Test TB32	110	20	1,500	81.9
Crash 1	103	16	1,100	34.2
Crash 2	115	21	1,340	87.8
Crash 3	119	15	955	34.9
Crash 4	95	23	1,040	55.3
Crash 5	119	22	1,625	124.6
Crash 6	105	20	1,400	69.7
Crash 7	95	16	1,445	38.2
Crash 8	114	17	985	42.2
Crash 9	105	12	890	16.4
Crash 10	102	25	1,300	93.1
Crash 11	84	11	1,255	12.4
Crash 12	125	20	1,420	100.1

#### 2.—In-depth crash investigations

The collection of data, investigation, reconstruction and analysis of each one of the 12 run-off crashes against an “N2 level” MRSB included in this article has been carried out by a special team of crash research experts since 2010 in the Spanish province of Valladolid. All these crashes are single vehicle crashes, involved injured people (otherwise the police did not report them to us), happened on any type of non-urban road (belonging to national, regional or provincial road administrations), and were analyzed after the expert accident research team had received the notification of the crash from the police, moved immediately to the scene and gathered all the necessary data for the reconstruction (*scene*: sketches, prints, vestiges, interviews of non-injured occupants and witnesses; *vehicle*: type, dimensions, weight, deformations, intrusions, …; and specially information concerning the *metal barrier*: type, dimensions, deformations, contact points,…). These types of investigations are called *“in-depth investigations”* because the special team of crash researchers gathers a lot of detailed information. They are not easy to perform since they require the collaboration of traffic police and a quick displacement of full data collection equipment at a very early stage of the crash investigation and they are economically very expensive. These are the main reasons for the low number of in-depth real crashes in this study. Future research may focus on analyzing more accidents, including not only passenger cars, but also heavy goods vehicles or buses. Fully detailed information coming from these accidents is not public (due to confidentiality reasons), but is incorporated into a private accident database for road safety studies [9–11] and this is taken as the basis for the development of new regulations.

### Variables

There are two types of variables considered in this study. The first set comes from the variables considered in the crash tests (Table 1), and the second one from those variables collected during the in-depth investigations. These variables are summarized in Table 2, and their definitions are detailed below.

**Table 2:** List of variables considered in this study based on crash tests for MRSB from standard EN-1317-2, and on in-depth investigations of real crashes.

Crash Tests Variables	In-Depth Investigation Variables
Impact speed against safety barrier	Exit Box
Impact angle	Barrier Safety
Total vehicle mass	Relative Orientation Impact Angle
Lateral kinetic energy	

The list of variables (Table 2) included in this study related to the parameters detailed in the EN-1317-2 standard and also measured in the 12 run-off crashes investigated in-depth are:

- Impact speed against safety barrier “v” (V1): Vehicle speed at the moment the vehicle impacts against the metal barrier (km/h).
- Impact angle “α” (V2): Angle between the velocity vector of the center of gravity of the vehicle and the barrier face at the moment of impact (^o^).
- Total vehicle mass “m” (V3): Total weight of the vehicle, including occupants and load (kg).
- Lateral kinetic energy “E” (V4): This variable is defined as:

$$E=\frac{1}{2}*m*{\left(v*\sin\alpha \right)}^{2}$$ (Eq 1)

The EN-1317-2 standard includes an additional variable describing the type of vehicle (passenger car, bus, rigid or articulated heavy goods). Since, in this study, all crashes considered are passenger car crashes, this variable is not further considered here.

**Fig 1:**
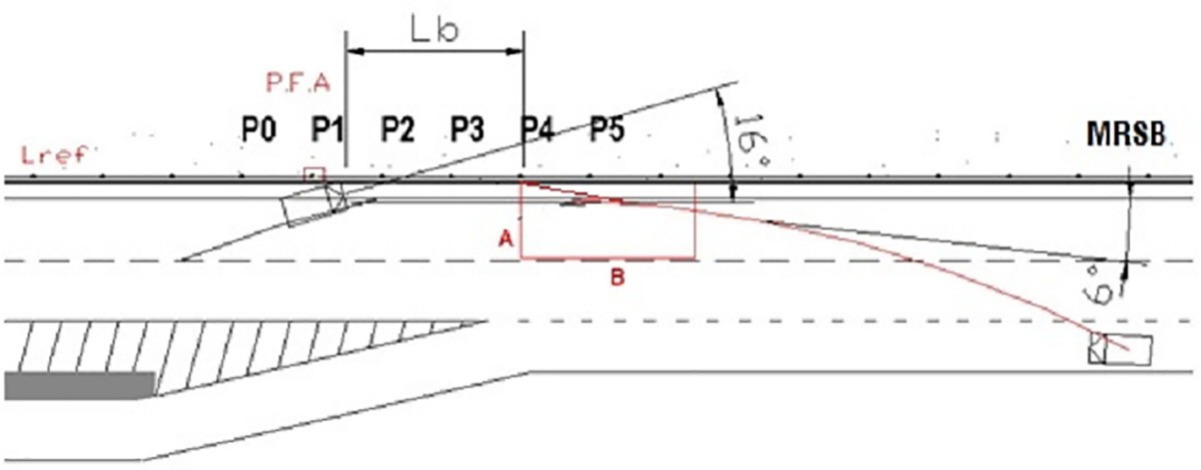
*Exit Box* in a collision in which the vehicle crashes with an impact angle of 16º, stops contacting the barrier at pole P4 with an exit angle of 6°, and crosses lateral line A of the exit box (“safe behavior”).

A second list of variables analyzed in this article is related to the moment just before and after the impact (either for the tests or for the crashes) to analyze the behavior of the MRSB:

- “Exit Box”: EN1317 defines this variable to consider post impact vehicle trajectories as “acceptable”. The exit box is characterized by a parallelogram whose sides are A (2.2 meters plus the width of the vehicle plus 16% of the length of the vehicle) and B (10 meters for the case of passenger cars). This parallelogram is virtually positioned from the last contact point between the vehicle and the RRS (see Fig 1).

The RRS will be “safe” if all the vehicle’s wheels cross the last line A (this means the vehicle must leave the box at a relatively low angle).

In the opposite case (the vehicle wheel tracks re-cross the line B), the barrier is considered as “not safe” (see Table 3), as well as in the cases when the vehicle has overrun the RRS.

**Fig 2:**
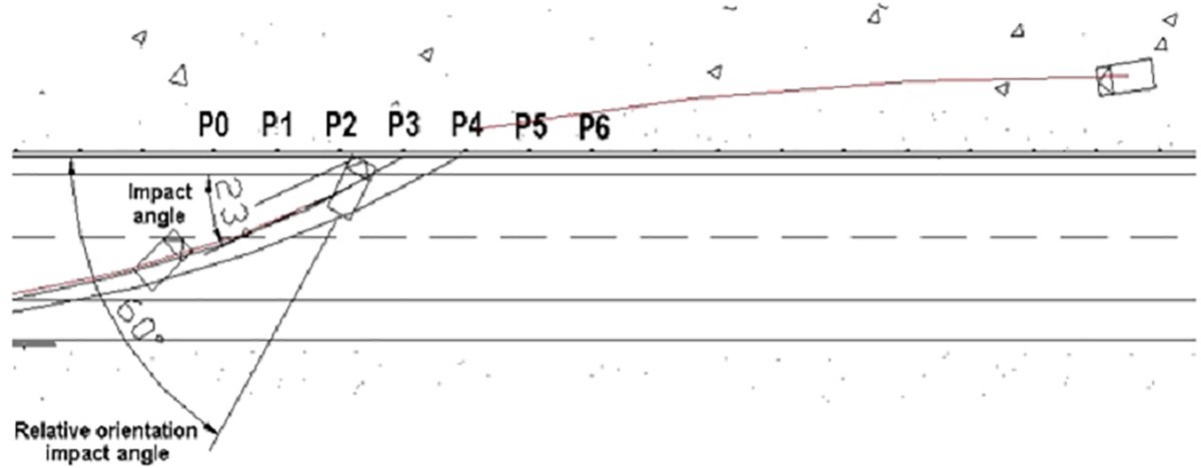
*“Impact angle* and *Relative orientation impact angle* in the analysis of one of the crashes.

- “Barrier Safety”: This variable records whether the barrier has behaved in a safe way during the crash (i.e., the vehicle has not crossed the barrier and has not crossed line B of the exit box in Fig 1) or not.
- *“Relative Orientation Impact Angle”*: Angle between the longitudinal axis of the vehicle and the safety system at the same point of the barrier. Even though, during all the tests defined in EN-1317-2, the *“impact angles”* coincide with the *“relative orientation impact angles”*, in real crashes, this equivalence does not exist. To measure these two different angles in the scene, it is very important to distinguish correctly the vehicle prints on the carriageway and those belonging to each vehicle wheel (see Fig 2). For example, if a *relative orientation impact angle* is equal to 146°, this would mean the vehicle was in a reverse motion before impacting the barrier, although the *impact angle* could be, for example, 23°.

**Table 3:** “Impact angle” and “Relative orientation impact angle” in the 12 in-depth analyzed real crashes.

Crash Number (r_i_ in Fig 3)	Impact angle (degrees)	Relative orientation impact angle (degrees)	Does the vehicle cross lateral line B of the ‘Exit Box’?	Is the Barrier safe?: 1 –Yes. 2- No, the vehicle crosses the barrier or the vehicle crosses the Exit Box (side B).
Crash 1 (r1)	16	16	No	1- Yes.
Crash 2 (r2)	21	19	Yes	2- No.
Crash 3 (r3)	15	146	Yes	2- No.
Crash 4 (r4)	23	60	Not applicable (vehicle crosses the barrier)	2- No.
Crash 5 (r5)	22	103	Yes	2- No.
Crash 6 (r6)	20	47	Not applicable (vehicle crosses the barrier)	2- No.
Crash 7 (r7)	16	37	Not applicable (vehicle crosses the barrier)	2- No.
Crash 8 (r8)	17	58	Yes	2- No.
Crash 9 (r9)	12	26	No	1 –Yes.
Crash 10 (r10)	25	101	Not applicable (vehicle crosses the barrier)	2- No.
Crash 11 (r11)	11	237	Yes	2- No.
Crash 12 (r12)	20	66	Not applicable (vehicle rests in the barrier)	1 –Yes.

### Study design and sample selection

A set of 12 run-off crashes against N2-level MRSB have been analyzed after receiving the notification of the crash from the police, moving immediately to the scene and gathering all the necessary information. These collisions took place from 2010 onwards in the Spanish province of Valladolid, outside urban areas and mainly on main roads or highways.

These have been the selection criteria for the crashes:

- Crashes with a single vehicle involved, independently of the vehicle type. As has already been mentioned, all the vehicles in the study were passenger cars.
- Run-off with later impact against a road safety barrier, irrespective of the system type. In the study, all the RRS analyzed were N2-level MRSB, which is the most common type of barrier on the roads.
- Only crashes with injured people were considered. Casualties involved in the 12 crashes were only slightly injured, except for one case where the driver sustained an intracranial injury scored as AIS 3 (using the Abbreviated Injury Scale [12]).

The analysis from these 12 run-off crashes has been compared with information coming from the standard EN 1317–2. This standard serves as the framework for the CE (Conformité Européenne) marking of road safety systems such as MRSB. In order to receive the CE marking, these road safety systems must be tested following the requirements described in this standard (variables and acceptance criteria), so they can be implemented within Europe once they have complied with this norm.

### Data analysis and statistical methods

#### 1.—Analysis of the real crashes and test cases

The main objective of this study is to achieve a better insight into the behavior of vehicles during a real impact against an MRSB with the aim of helping to improve current test standards to assess, in general, RRS behavior. For this, real crashes studied in-depth have been compared with the standard tests. Although there are two standards for evaluating MRSB in the world (EN-1317-2 [7] and MASH [8]), taking into account the fact that the 12 in-depth crashes happened in Spain, the standard used to compare the real crashes and the configuration standard tests has been the European one, EN-1317-2. As the investigations of real crashes have been carried out specifically for passenger cars, the relevant test configurations to be considered in this study are TB11, TB21, TB22, TB31 and TB32 (see Table 1).

The statistical method used for this comparison is the so-called Multidimensional Scaling (MDS [13]), which is a way of visualizing the level of similarity of individual cases in a dataset. It is a form of non-linear dimensionality reduction. An MDS algorithm aims to place each object in *D*-dimensional space such that the between-object distances are preserved as nearly as possible. Each object is then assigned coordinates in each of the *D* dimensions. The number of dimensions of an MDS plot *D* can exceed *2* and is specified a priori. Choosing *D = 2* optimizes the object locations for a two-dimensional scatterplot. In this application, the method tries to find the structure of a set of distance measurements between different cases (between crash cases and test cases). This is possible by the allocation of the crash and test observations to specific positions in a conceptual space (usually two or three dimensions). The dimensions of this conceptual space are sometimes interpretable and can be used to better understand the data. An interesting advantage of the use of Multidimensional Scaling (MDS), with respect to other possible approaches, is that it can be generally applied to cases even where only distances between crashes are available. Therefore, many distances (based on numerical, ordinal or even categorical variables) can be also applied if needed.

In this study, the initial data belong to a four dimension space (original space), as the four variables used for the comparison are: Impact speed (“v” or V1), Impact angle (“α” or V2), Total vehicle mass (“m” or V3) and Lateral Kinetic Energy (“E” or V4). Starting from the Euclidean distance *d(ij)* defined in that four dimension space between two crashes ‘*i*’ and ‘*j*’ as $d(ij)=\sqrt{{\left(v_{i}-v_{j}\right)}^{2}+{\left(\alpha_{i}-\alpha_{j}\right)}^{2}+{\left(m_{i}-m_{j}\right)}^{2}+{\left(E_{i}-E_{j}\right)}^{2}},$ the multidimensional scaling method allows us to work in a new, two dimensional space (conceptual space) with only a small loss of information, as will be seen later. In this new conceptual space, it is possible to visualize the proximity between each crash and each test in an easier way. Prior to the application of this procedure, the original data should be suitably standardized, so that the original variables can be combined independently of their measurement units and their different dispersions.

#### 2.- Analysis of the crash dynamics

The second part of this study is focused on the analysis of the vehicle dynamics before and after the impact against the MRSB, so as to be able to explain why the MRSB can perform poorly in situations that are not far from the standard test conditions. To achieve this aim, the differences between *“Impact angle”* and *“Relative orientation impact angle”* and its relation with the safety performance of the barrier have been studied in the real crashes and the final trajectory of the vehicle after the crash *(“Exit Box”)* using descriptive and inferential statistical procedures.

## Results

### 1. - Comparison between real crashes and test configurations proposed in the standard

**Fig 3:**
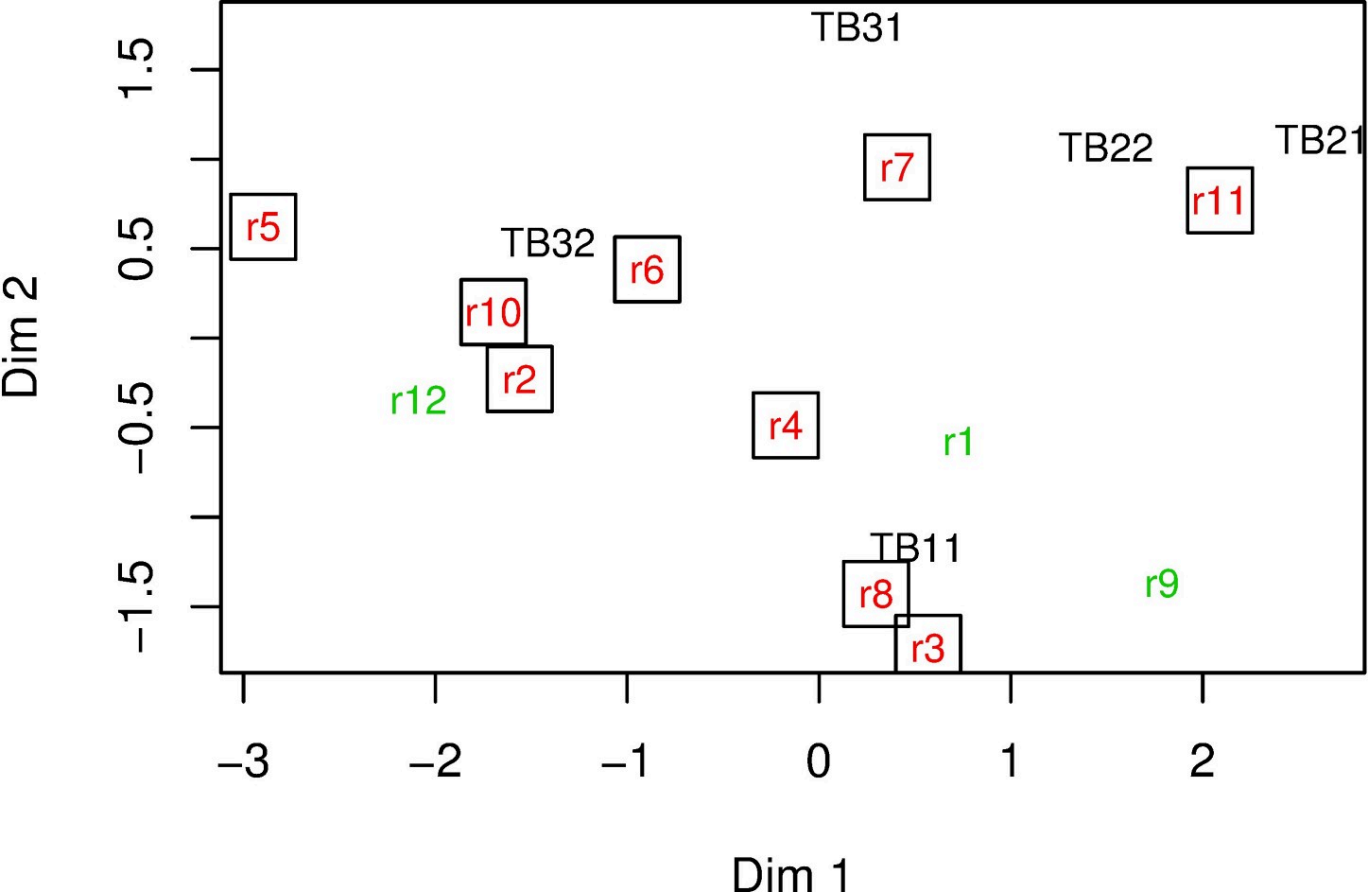
Representation of crashes and the tests proposed in the EN-1317-2 in the (2-dimensional) conceptual space provided by the multidimensional scaling method (MDS). In green, crashes considered as “safe” due to the behavior of the MRSB and in boxed red the crashes considered as “unsafe”.

To perform the MDS, the distribution free software R [14] has been used. Fig 3 shows the representation of the real crashes and test cases in the conceptual space, obtained through the application of the MDS methodology. To understand completely the graphic, it should be noted that the cases from 1 to 12 refer to the real world crashes detailed in Table 1, while the tests are represented as TBxy.

It can be observed that most of the crashes can be assigned to the “TB11 test” (cases r1, r3, r4, r8 and r9), and “TB32” (cases r2, r5, r6, r10 and r12). Remember that these two tests are the relevant ones for the N2 containment level. There is one collision (case 7) equally close to “TB22” and “TB31” and one more equally comparable to”TB21” and “TB22” (case 11). Thus, due to the equidistance of case r7 and case r11 to their closest tests, these two crashes cannot be assigned clearly to any of them.

The axes in the plot can also be given an interpretation. It can be said that dimension 1 is mainly related to variables V2 “impact angle” and V4 “lateral kinetic energy”, as it opposes tests TB32 (on the left of the graph) and TB21 (on the right side), which are the tests with the lowest and highest values for both variables V2 and V4, respectively (see the values for the tests in Table 1). As for dimension 2, this dimension is mainly related to variable V3 “total vehicle mass” as it opposes the tests with more than 1000 kg above the 0 value with TB11 (below the 0 value in this axis) which is the only test with a mass under 1000 kg.

It may be considered that analyzing data after reducing the number of dimensions might cause a loss of information. However, the R-squared value of the MDS procedure (*R*^*2*^ *= 0*.*92*) shows that less than 8% of the information is lost by mapping the original four dimensional dataset to that conceptual two dimensional space and, therefore, the representation reflects the original distances very accurately. For this reason, there is no need to consider a higher number of dimensions to represent the distances between the original data.

### 2. - Relation between the absolute difference between “Impact angle” and “Relative orientation impact angle” and “Exit Box”

From the previous analysis, it can be observed (see Fig 3 and Table 3) that, although the barrier has met the EN-1317-2 standard and the real crashes are close to the standard test, the vehicle trajectory frequently crosses the lateral line B of the *“Exit Box”*, or the vehicle even crosses the barrier and consequently the barrier is not performing safely. For example, it can be seen that crashes r6, r10 and r2 are the closest ones to the standard “TB32” and r8 and r3 are close to “TB11”. This means that there may be additional variables not included in the tests that can play an interesting role in crashes.

**Fig 4:**
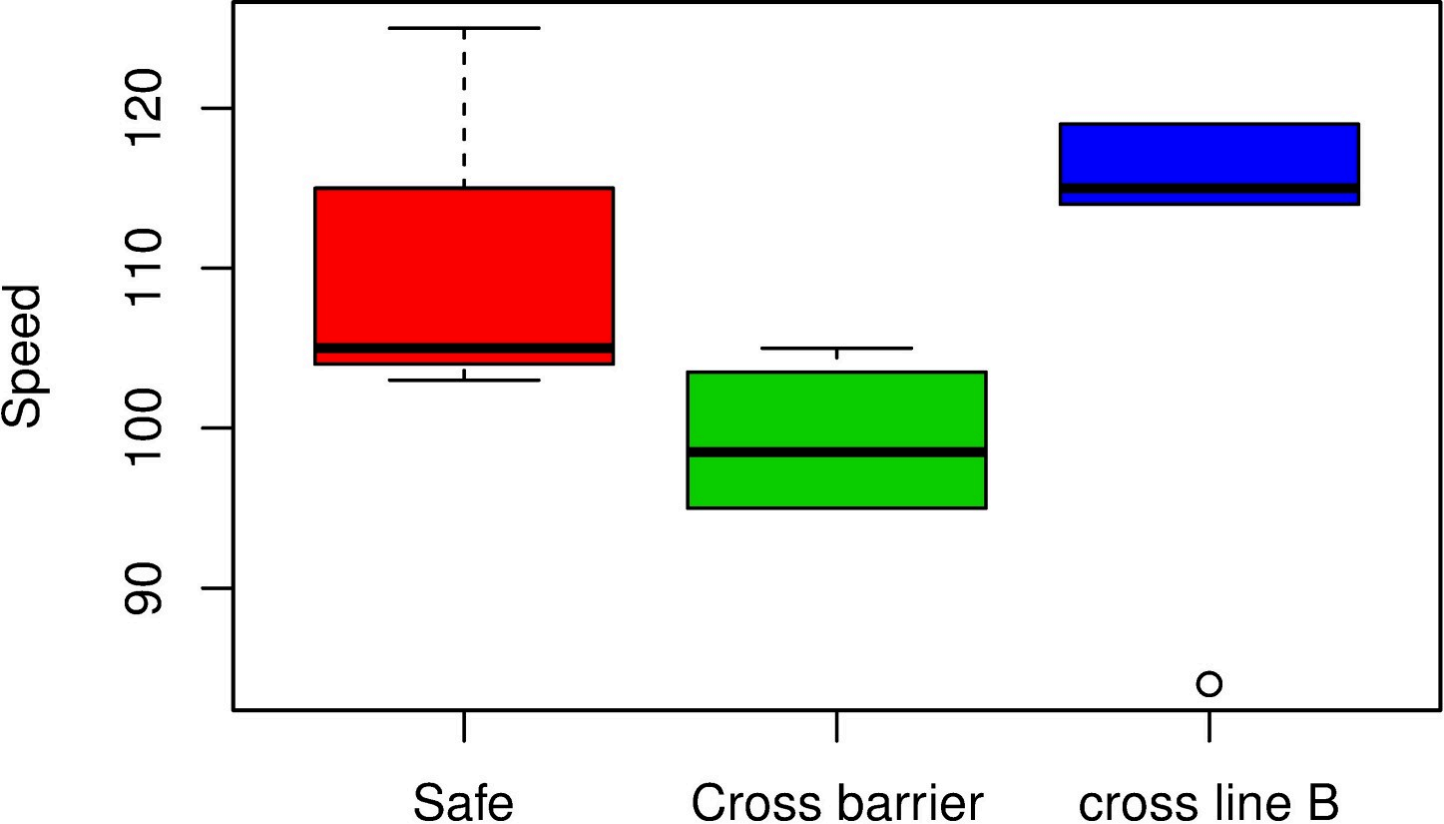
Multiple box-plot showing that there is not a clear direct relation between “Speed” and “Safety” since “Speed” values for “Safe” cases are intermediate between those registered for “unsafe” cases (“Cross barrier” and “cross line B”). The limits of the boxes are the quartiles of the corresponding variables.

**Fig 5:**
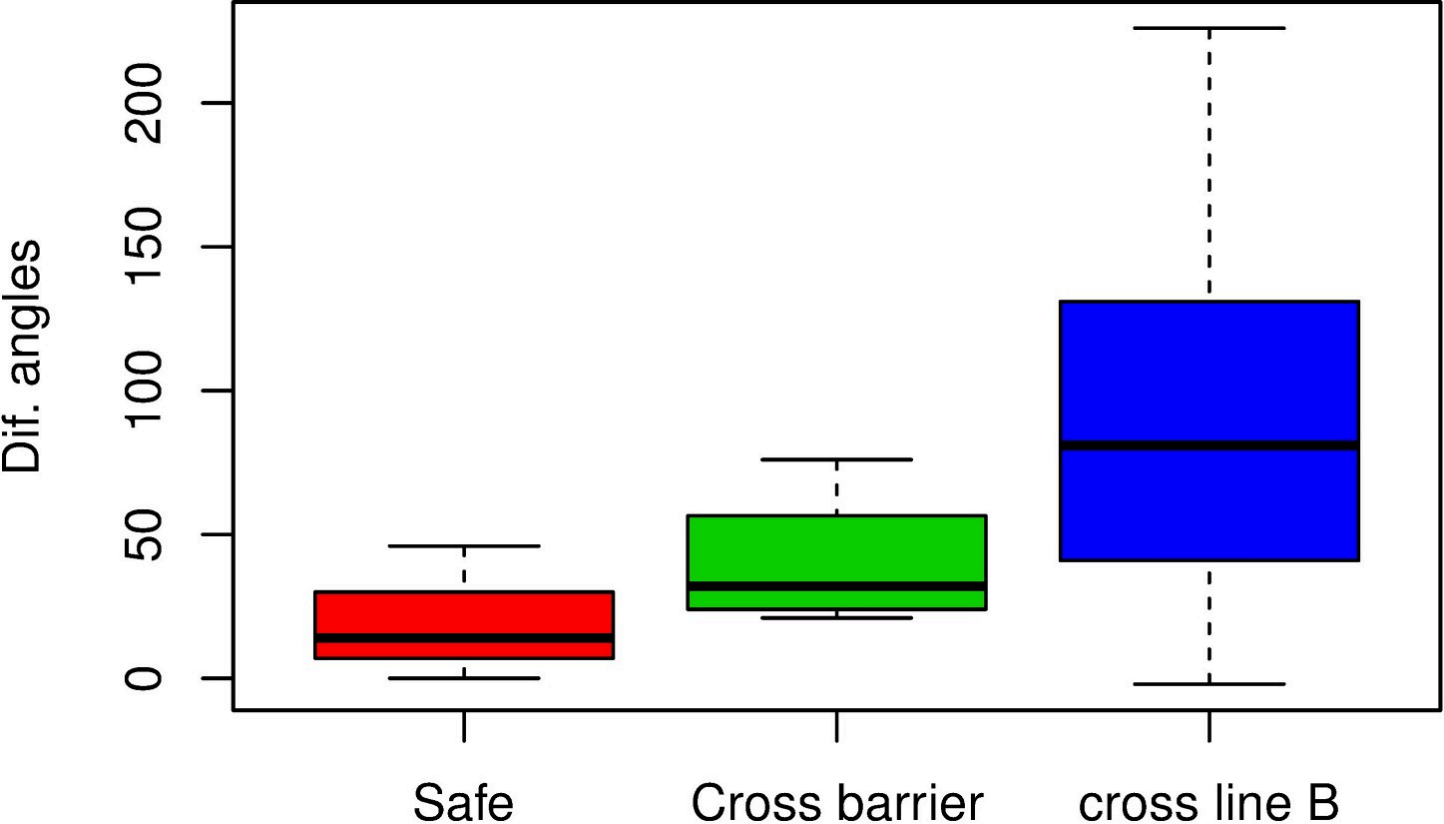
Multiple box-plot showing a relation between “Difference between angles” (the absolute difference between “Impact angle” and “Relative orientation impact angle”) and “Safety” as the values for “Difference between angles” are generally lower for “Safe” cases than for “Unsafe” (“Cross barrier” and “cross line B”) ones. The limits of the boxes are the quartiles of the corresponding variables.

Table 3 offers the values of the variables, *“Impact angle” and “Relative orientation impact angle”*, for the real crashes together with variables describing the safety performance of the barrier (the barrier is safe when the vehicle does not cross the barrier or the line B of the exit box). The values of the *“Impact angle”* already appeared in Table 1, but are included again in Table 3 so that they can be easily compared with the values of the *“Relative orientation impact angle”*. The full Table containing the data on the real crashes can be found in S1 File. Fig 4 shows that there does not seem to be a direct relation between safety and speed, since speed values for safe cases are intermediate between those registered for unsafe cases. On the other hand, Fig 5 exposes a possible relation between high absolute differences between the variables *“Impact angle” and “Relative orientation impact angle”* and the situations in which the vehicle trajectory crosses the lateral line B of the *“Exit Box”*, or the vehicle even crosses the barrier, as the values for the absolute difference between angles are generally lower for safe cases than for unsafe ones. We have considered absolute differences as the small sample size did not allow to reasonably account for differences due to the orientation towards the barrier. Orientation is sure an interesting point to be further investigated when a larger sample is available.

To strengthen our conclusions, we performed two logistic regression analyses relating the response variable *“Safety”* (last column in Table 3) with *“Speed”* and the absolute difference between *“Impact angle”* and *“Relative orientation impact angle”* as explanatory variables, respectively. Logistic regression [15] is a widely used statistical technique that allows to evaluate the relationship between a dichotomous response variable (such as *“Safety”*) and a set of explanatory variables. The results of these analyses can be found in Table 4. Although care should be taken due to small sample size, results in Table 4 show that absolute difference between *“Impact angle”* and *“Relative orientation impact angle”* is significant at the 0.1 level meaning that we are 90% sure that this variable has an impact on “Safety”, while this does not happen when “Speed” is considered as explanatory variable as its p-value (0.4507) is much higher than that level. Notice that this does not mean that “Speed” does not play a role in safety but that that factor is already taken into account in the standard tests so that, as the real crashes considered are not far from the tests (as the MDS analysis shows), “Speed” cannot explain further variability. On the other hand, *“Relative orientation impact angle”* is not measured in the standard tests but its absolute difference with *“Impact angle”* plays a role in safety as exposed in the results in Table 4.

**Table 4:** Logistic regression deviance analysis results with “Safety” as response and “Speed” and absolute difference between “Impact angle” and “Relative orientation impact angle” as explanatory variables.

		“Speed” as explanatory		Absolute difference between *“Impact angle”* and *“Relative orientation impact angle”* as explanatory	
*Source*	*Df*	*Deviance*	*P-Value*	*Deviance*	*P-Value*
Model	1	0.568984	0.4507	2.72243	0.0989
Residual	10	12.9271		10.7736	
Total (corr.)	11	13.496		13.496	

It should be remarked that prior to the analysis of any real crash, it would seem intuitive to think that, when a vehicle runs off (with a later collision against an MRSB), the *“Relative orientation impact angle”* coincides with the *“Impact angle”* (if the driver is not able to react), or is close to it. However, although the number of crashes investigated has only been 12, the analysis shows that, in most of these cases, the *“Relative orientation impact angle”* has been very different from the “Impact angle”. Therefore, in all these real crashes, either for one cause (run off through its lane and try to return to the carriageway after sudden maneuver with later impact against the barrier) or another (crossing from a lane to another one and running off), the final trajectory of the vehicle just at the moment of the impact was different to the relative orientation of the vehicle with regard to the barrier.

## Discussion

The use of statistical methods (MDS) allows us to better understand how close real crashes are to standard tests, using several impact variables at the same time. The collection of detailed information from real impacts can be useful, especially if these variables are treated together and compared with those coming from the tests. In this paper, it has been argued that most of the real crashes studied can be assigned to the impact tests necessary to obtain an “N2 containment level” for an RRS (meeting “TB11” and “TB32” tests). This suggests that these two types of tests are very interesting in run-off crashes, as they represent many usual real crashes.

However, although the barriers installed on the roads passed the standard tests, in many of these real cases the barriers did not properly contain the impact received. In this study, we have observed that, in many of these cases, the variables *“Impact angle”* and *“Relative orientation impact angle”* are different, and the most important conclusion is that, in the situations in which the *“Relative orientation impact angle”* is very different from *“Impact angle”*, the vehicle is not redirected into the safety area (*“Exit Box”*), and this results in an “unsafe” behavior of the MRSB. This is the main reason why, although the number of analyzed crashes in this study is low, it is suggested that it could be interesting to include *“Relative orientation impact angle”* as a variable to be controlled in new versions of the EN-1317-2 standard, or even other similar standards, with the objective of helping to prevent injuries in crashes.

In order to look for a mechanistic explanation based on vehicle dynamics for the interest of the *“Relative orientation impact angle”*, notice that, if the vehicle is not rotating at the moment of the impact, the difference between the two angles is equal to zero. The tests performed on the barriers are performed under that condition. What we are finding here is that those conditions are not the ones under which most of the crashes happen, as there is usually a rotation movement in the car at the moment of impact (possibly due to the driver trying to avoid collision and redirecting the vehicle to the road). The higher the yaw rotation is, the bigger the difference between the angles is. Our results suggest that this higher difference is associated to the barrier not working properly. It may be argued that the yaw rotation might have been considered in this study, instead of the difference between the angles. However, accident reconstruction methods [16, 17] are based on measuring skid marks, vehicle positions, impact deformations (both in the infrastructure and in the vehicles) and all the vestiges that an accident researcher can assess after the accident. Even with this information, it is very difficult to obtain some velocities, such as, for instance, yaw velocities. The usual way to measure yaw rotation is to use an accelerometer in the vertical axis of the vehicle, but this instrument is not incorporated into standard vehicles.

A discussion on the low sample size considered in this article is also convenient. As already mentioned, there are two main reasons for the low number of real crashes considered in this study. The first of them is that it is not easy to perform the so-called “in-depth investigations” of crashes made here, as the data collector has to be contacted by the traffic police at a very early stage of the crash investigation and the full equipment has to move to the crash site in a very short time before skid or breaking marks disappear. The other reason is obviously economic, as the cost of this in-depth data collection is extremely high. Consequently, as we believe that our conclusions may be of help to prevent injuries, we consider that the results set out in this study are valuable enough to be reported in spite of the sample size, as it might take a long time to be able to achieve a sample size large enough to be able to consider other methods and there is no guarantee that there would be enough money in the future to do so.

In any case, we would like to stress that our intention is to carry on collecting these in-depth data, not only to apply our MDS methodology with a higher number of cases, but also to be able to reinforce the conclusions obtained with the inferential techniques (logistic regression) that we have considered in this study and to consider other possibly interesting questions such as, for example, the orientation of the vehicle towards the barrier.

Notice also that, in this study, we have only considered run-off crashes with passenger cars involved. It would obviously be interesting to consider other types of vehicles (buses, heavy goods vehicles …). An increased number of cases would also allow us to obtain results for the rest of vehicle types. One advantage of the study presented in this article is that the multidimensional scaling methodology considered here can be used without any change to easily incorporate these other crashes into the analysis.

## Conclusions

While the conditions under which the standard tests are performed to assess the impact behavior of MRSB may be close to those of real crashes, we have seen that the set of variables considered for the tests seem not to be complete enough to properly describe the real situations, resulting in an unsafe impact behavior of the road safety barriers. The set of real crashes analyzed here suggests that it could be interesting to include the *“Relative orientation impact angle”* as a variable to be controlled in new versions of the EN-1317-2 standard, with the objective of helping to prevent injuries in run-off road crashes.

The need to enlarge the real accident data set is also clear, so as to be able to consider other inferential methods that may allow stronger statistical significance to be achieved for the conclusions. This larger sample size, that will be neither easy nor cheap to obtain, would also allow the multivariate methodology used here to be extended to crashes where other vehicles, apart from passenger cars, are involved. We also expect this larger sample size to reinforce the conclusions obtained here which, in our opinion, if taken into account, may be of help in preventing injuries in future crashes.

## Supporting information

S1 File S1 File contains the data on the real crashes considered in the paper. (PDF)
