# Supplementary material for: Analysis of real crashes against metal roadside barriers
Source: PLoS One. 2019 Feb 4;14(2):e0211674. doi: 10.1371/journal.pone.0211674 (PMC6361448; doi:10.1371/journal.pone.0211674)
Supplement: S1 File — (PDF) [file pone.0211674.s001.pdf]

Crash Number	Impact speed (km/h)	Impact angle (degrees)	Total vehicle mass (kg)	Lateral Kinetic Energy (kJ)	Relative orientation impact angle (degrees)	Does the vehicle cross lateral line B of the 'Exit Box'?	Is the Barrier safe?:<br><br>1 – Yes.<br>2- No, the vehicle crosses the barrier or the vehicle crosses the Exit Box (side B).
Crash 1	103	16	1,100	34.2	16	No	1- Yes.
Crash 2	115	21	1,340	87.8	19	Yes	2- No.
Crash 3	119	15	955	34.9	146	Yes	2- No.
Crash 4	95	23	1,040	55.3	60	Not applicable (vehicle crosses the barrier)	2- No.
Crash 5	119	22	1,625	124.6	103	Yes	2- No.
Crash 6	105	20	1,400	69.7	47	Not applicable (vehicle crosses the barrier)	2- No.
Crash 7	95	16	1,445	38.2	37	Not applicable (vehicle crosses the barrier)	2- No.
Crash 8	114	17	985	42.2	58	Yes	2- No.
Crash 9	105	12	890	16.4	26	No	1 – Yes.
Crash 10	102	25	1,300	93.1	101	Not applicable (vehicle crosses the barrier)	2- No.
Crash 11	84	11	1,255	12.4	237	Yes	2- No.
Crash 12	125	20	1,420	100.1	66	Not applicable (vehicle rests in the barrier)	1 – Yes.
